# Patient selection, clinical outcomes, associated healthcare utilization, and cost-effectiveness of basivertebral nerve ablation for the treatment of vertebrogenic low back pain: A narrative review^1^

**DOI:** 10.1016/j.xnsj.2025.100788

**Published:** 2025-09-08

**Authors:** Reza Ehsanian, Jordan A. Buttner, Byron Schneider, Zachary L. McCormick

**Affiliations:** a Division of Pain Medicine, Department of Anesthesiology & Critical Care Medicine, University of New Mexico School of Medicine, Albuquerque, NM, United States; b University of New Mexico School of Medicine, Albuquerque, NM, United States; c Department of Physical Medicine and Rehabilitation, Vanderbilt University Medical Center, Nashville, TN, United States; d Department of Physical Medicine and Rehabilitation, University of Utah School of Medicine, Salt Lake City, UT, United States

**Keywords:** Low back pain, Chronic pain, Cost-effectiveness analysis, Intervertebral disc degeneration, Patient selection, Basivertebral, Basivertebral nerve ablation, Zygapophyseal joint, Treatment outcome, Visual Analog Scale

## Abstract

**Background:**

Chronic low back pain (cLBP) represents a significant burden to global health, with a prevalence projected to reach 843 million individuals by 2050. Vertebrogenic cLBP, a distinct phenotype, is mediated by nociception transmitted through the basivertebral nerve. Advances in basic and translational science have established clinical imaging biomarkers of vertebrogenic cLBP, such Type 1 and 2 Modic changes, to more reliably identify this condition. Additionally, medical technology advances have provided the ability to selectively disrupt pain signaling from painful vertebral endplates by interosseous basivertebral nerve ablation (BVNA). The objective of this review is to highlight appropriate patient selection, clinical outcomes, associated healthcare utilization, and cost-effectiveness of BVNA in the treatment of vertebrogenic cLBP.

**Methods:**

PubMed, EMBASE, and Google Scholar databases were queried for articles published before September 2024. Two authors reviewed references for eligibility, extracted data, and appraised the quality of evidence.

**Results:**

Patient selection criteria include the presence of Type 1 or Type 2 Modic changes on MRI in the context of clinical suspicion of anterior element spinal pain based on clinical evaluation. BVNA was found to result in clinically significant and sustained pain relief and functional improvements in individuals with vertebrogenic cLBP. Randomized controlled trials and systematic reviews demonstrate long-term efficacy, with clinically meaningful benefits sustained up to 5 years postprocedure. Healthcare utilization analyses indicate that BVNA significantly reduces low back pain-related healthcare utilization, opioid use, and surgical intervention rates. Economic analysis indicates that BVNA is cost-effective when compared to conventional management of vertebrogenic cLBP.

**Conclusions:**

In appropriately selected patients, the overall body of evidence demonstrates that BVNA is an effective and durable treatment for vertebrogenic cLBP.

## Introduction

Chronic low back pain (cLBP) represents a significant burden to global health, affecting an estimated 619 million people in 2020, with projections reaching 843 million by 2050 [1]. Historically, the treatment of cLBP has been hampered by challenges in identifying specific pain sources and limited high-quality evidence supporting therapeutic options [2]. Advancements in understanding the biochemical, biomechanical, and pathophysiological underpinnings of spinal pain conditions have enabled the identification of cLBP patient subgroups with distinct patterns of damaged anatomical structures, chronic inflammation, and edema [[2], [3], [4], [5]]. Among these subgroups of cLBP, the vertebral endplate is distinct from other sources and can be diagnosed with reliability [2,6]. Individuals with vertebrogenic cLBP commonly present with midline-dominant low back pain with possible radiation into the gluteal region and exacerbation of pain with activity but not typically with lumbar extension positions [2,[7], [8], [9]]. Nociception from the vertebral endplate is transmitted to the central nervous system via the basivertebral nerve (BVN) which serves as the critical target for therapies [[10], [11], [12], [13]]. Intraosseous basivertebral nerve radiofrequency ablation (BVNA) was developed to achieve the goal of selective interruption of the BVN.

**Fig. 1:**
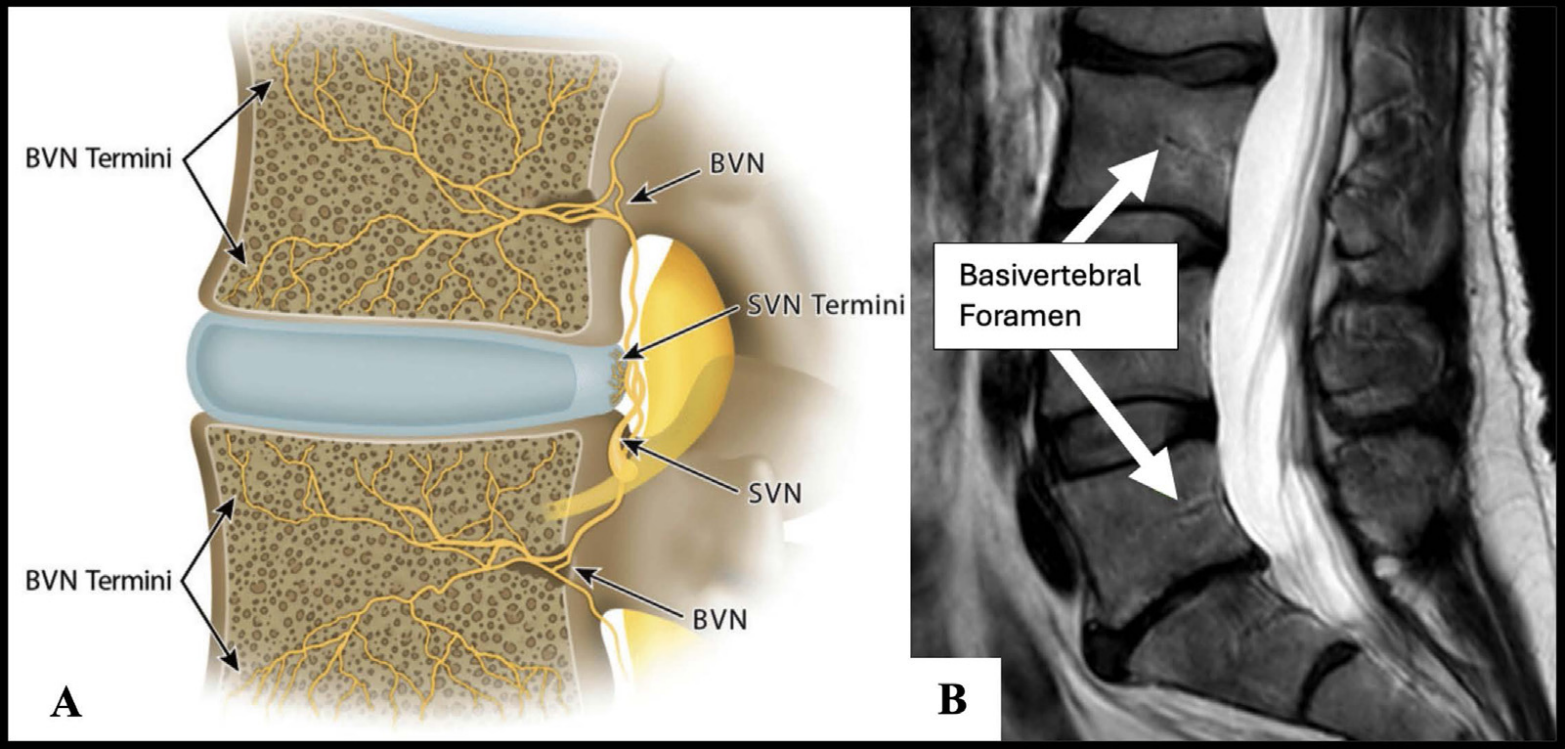
Anatomical distribution of basivertebral nerve. (A) Illustration depicting the distribution of the basivertebral nerve (BVN) branching from the sinuvertebral nerve (SVN) and innervating the vertebral body endplates. The nerve enters through the posterior vertebral body and extends toward the superior and inferior endplates, playing a role in vertebral pain transmission associated with degenerative disc disease. (B) Radiographic depiction of basivertebral foramen identified with white arrows at L3 and L5. (Image adapted from WikiMSK: Basivertebral Nerve (https://wikimsk.org/wiki/Basivertebral_Nerve) and Conger et al. (Vertebrogenic pain: a paradigm shift in diagnosis and treatment of axial low back pain), Licensed under Creative Commons Attribution-ShareAlike 4.0 International Deed).

BVNA is a minimally invasive, outpatient procedure that disrupts pain signals from the vertebral endplates to the central nervous system [10]. As the BVN is an unmyelinated nerve, it is thought to not regenerate or regenerate much more slowly than other peripheral nerves following radiofrequency ablation [10,11,14]. This may explain the greater durability of BVNA when compared to other peripheral nerve radiofrequency ablation procedures such as spinal facet joint denervation by medial branch nerve radiofrequency ablation (Fig. 1) [15,16].

**Fig. 2:**
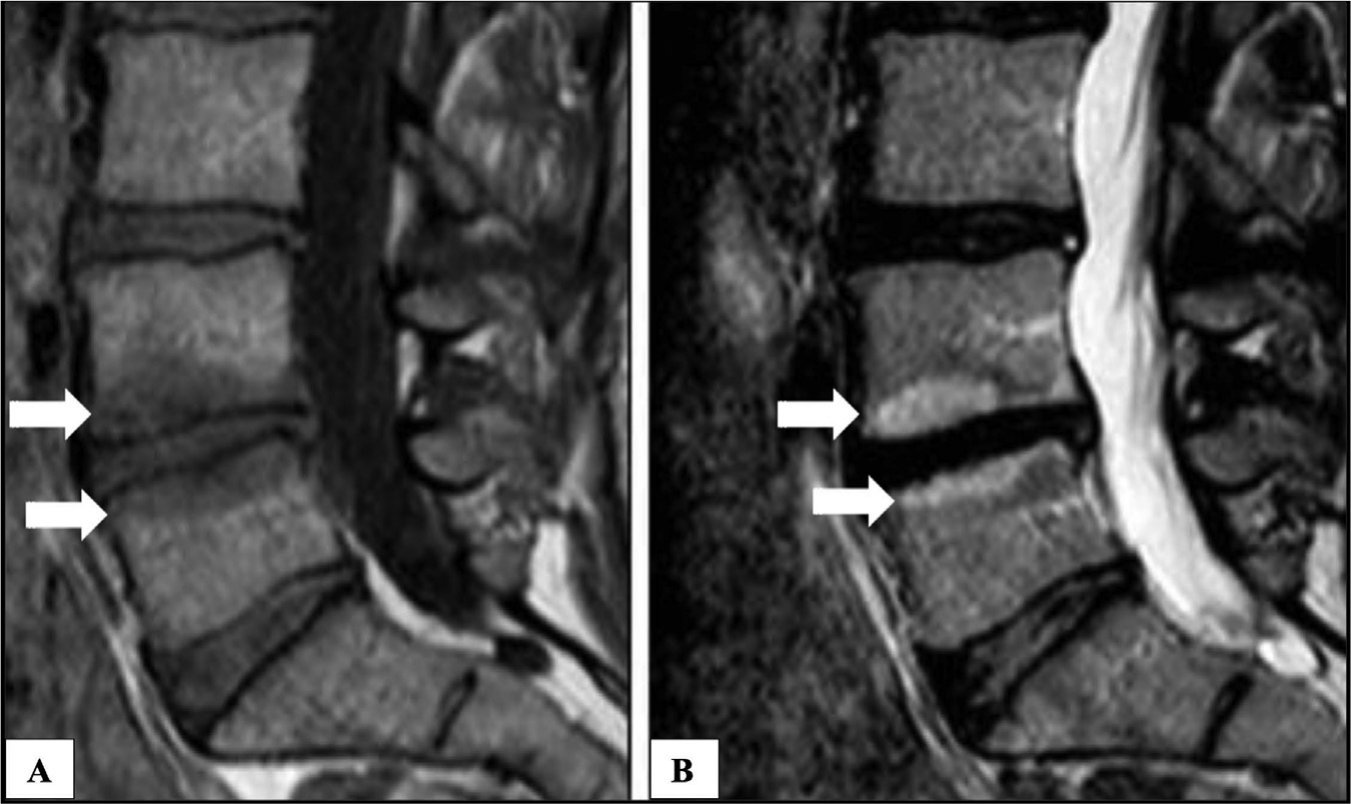
Type 1 Modic changes as visualized on magnetic resonance imaging. Sagittal T1-weighted (A) and T2-weighted (B) images of the lumbar spine showing hypointense signal on T1 and hyperintense signal on T2, indicative of bone marrow edema and inflammation at the vertebral endplates. These changes suggest an early degenerative process in the intervertebral disc and adjacent vertebral bodies. White arrows identifying bone marrow edema and micro-fractures around the L4-L5 intervertebral space. (Image adapted from Neurosurgery Education Wiki: Modic type 1 changes (https://neurosurgery.education/wiki/doku.php?id=modic_type_i_changes), Licensed under Creative Commons Attribution-ShareAlike 4.0 International Deed).

**Fig. 3:**
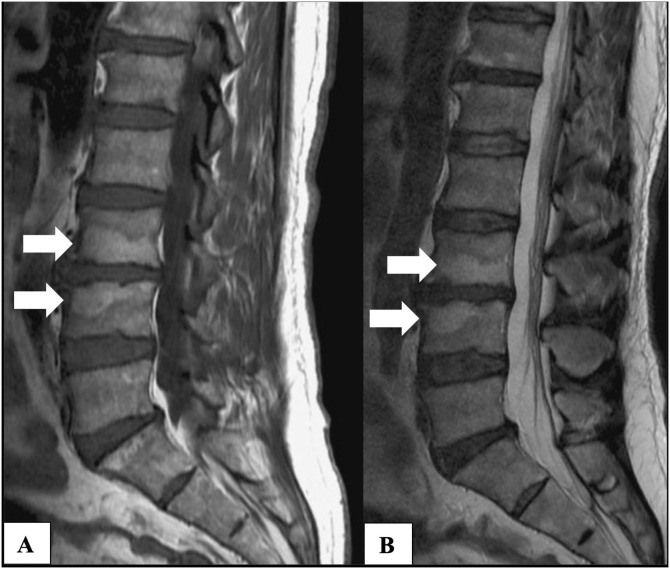
Type 2 Modic changes as visualized by magnetic resonance imaging. Sagittal T1-weighted (A) and T2-weighted (B) images of the lumbar spine showing hyperintense signal on T1 and isointense or mildly hyperintense signal on T2, indicative of fatty infiltration of the bone marrow at the vertebral endplates. These changes reflect a more chronic phase of degenerative disc disease compared to Type 1 Modic changes. White arrows identifying bone marrow with fatty replacement around the L3-L4 intervertebral space. (Image adapted from Modic Type II Endplate Changes by Frank Gaillard (https://radiopaedia.org/cases/modic-type-ii-endplate-change), Licensed under Creative Commons Attribution-ShareAlike 4.0 International Deed).

Patient selection for BVNA is also discrete from radiofrequency ablation for spinal facet joint denervation in that a diagnostic/prognostic block of the BVN is not feasible. Thus, other diagnostic criteria including imaging biomarkers of vertebrogenic cLBP are important for patient selection [2,[17], [18], [19], [20]]. Magnetic resonance imaging (MRI) can identify readily visible bone marrow lesions known as Type 1 and Type 2 Modic changes (Figs. 2 and 3, Table 2) that correlate with the presence of vertebrogenic cLBP [[21], [22], [23], [24], [25], [26], [27], [28], [29], [30], [31], [32], [33], [34]]. Of note, Type 1 Modic changes are over 4 times more prevalent in individuals 50 years of age or younger with low back pain compared to those who are asymptomatic [28]. Type 3 Modic changes, representative of sclerosis, have also been described but do not appear to be associated with cLBP (Table 2) [[35], [36], [37], [38], [39]].

**Fig. 4:**
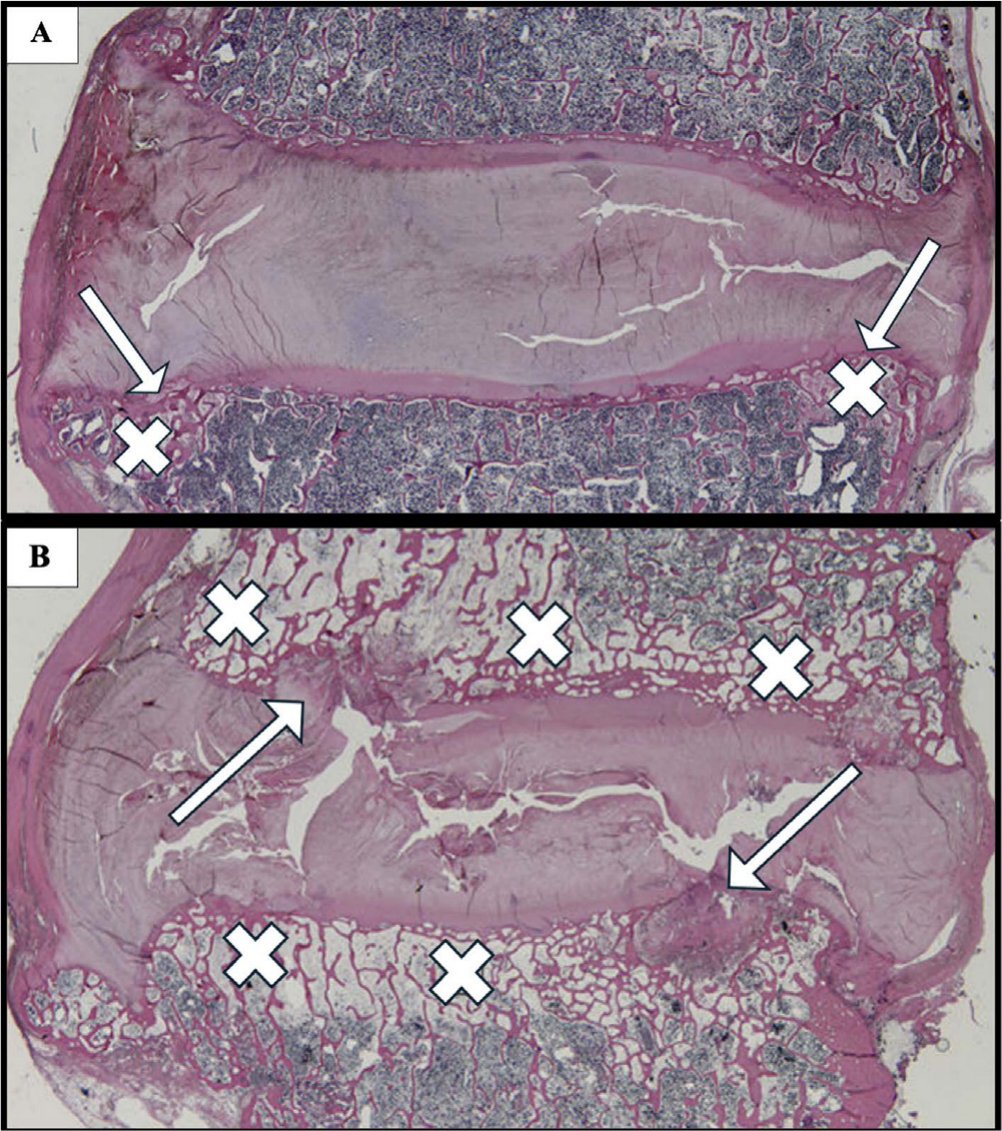
Histopathology of Modic disc changes. Representative microscopic image showing characteristic features of Modic changes in the intervertebral disc. Notable findings include disorganization of the annulus fibrosus, granulation tissue infiltration, and vascular proliferation. (A) Evidence of bone marrow changes and inflammatory cell infiltration is present, corresponding to Modic Type 1 changes. Crosses denote areas of characteristic fibrovascular changes and arrows mark irregular endplate borders. (B) Representative histopathological changes of Modic Type 2 changes with fatty replacement of normal marrow tissue (crosses) and fibrotic processes along endplate borders (arrows) representing chronic damage. Sample stained with H&E. (Image adapted from Pathobiology of Modic Changes by Dudli et al. (https://www.akot.com.ar/cokiba/cursos/2018/16_curso_oyt/files/nihms868217.pdf).

The clinical science of identifying patients with the phenotype of vertebrogenic cLBP, selecting appropriate patients for BVNA, and characterizing treatment outcomes continues to evolve. The utility of BVNA, as with any condition-specific intervention, hinges on accurate diagnosis. Furthermore, while characterization of pain and functional outcomes are important in any assessment of cLBP therapy, evaluation of healthcare utilization and cost-effectiveness are also vital in the larger picture of how a given treatment influences healthcare economics. As such, the aims of this review were to first characterize best practices in patient selection for BVNA, second, to catalogue published treatment outcomes associated with BVNA, and, third, to review published evidence related to low back pain-related healthcare utilization and cost-effectiveness associated with BVNA Figure 4.

## Methods

### Study selection

An extensive search of peer-reviewed literature databases including PubMed, EMBASE, and Google Scholar was conducted in September of 2024. Articles were limited to those published in English. Article type was not an exclusion criterion. Additional references were collected from the citations of reviewed articles, authoritative texts, and personal contacts with experts to ensure a comprehensive search process. Two authors independently reviewed eligible abstracts for relevance to the stated aims of this review and disagreements regarding inclusion were decided by a third author. This process was repeated with full-text reviews of articles to determine inclusion/exclusion of manuscripts.

The search terms for electronic database queries included terms such as “basivertebral nerve ablation,” “vertebrogenic pain,” “Modic changes,” “chronic low back pain,” “radiofrequency ablation,” “cost-effectiveness,” “clinical outcomes,” and “pain reduction.” A final search strategy was determined by entering a draft of the current review’s abstract and the above listed terms into the Medical Subject Headings (MeSH) on demand site (https://meshb.nlm.nih.gov/MeSHonDemand).

## Results and discussion

### Patient selection

Clinical trials with narrowly-defined patient selection criteria demonstrate high treatment responder rates following BVNA, particularly when compared to conventional treatments for cLBP [40,41]. Such inclusion/exclusion criteria (Table 1) may reduce eligibility to just 3% (95% CI: 1%−5%) of all cLBP patients without radicular symptoms [42]. To further optimize BVNA responder rates and utilization, multiple investigators have applied predictive modelling to additional demographic, clinical, and imaging factors beyond those captured within such trials.

**Table 1:** Inclusion and exclusion criteria for pooled cohort analysis of BVNA patient selection factors

Inclusion criteria
• Skeletal maturity. • Lack of response to ≥6 months of nonoperative chronic low back pain (cLBP) management. • Oswestry Disability Index (ODI) score ≥30. • Visual Analog Scale (VAS) score ≥40 mm.

Exclusion criteria
• Modic changes outside the L3–S1 levels. • Presence of radicular pain or symptomatic spinal stenosis. • Prior lumbar spine surgery (except discectomy/laminectomy >6 months prior). • Presence of metabolic bone disease. • History of spinal fragility or traumatic fracture. • History of spinal cancer. • Disc protrusion or extrusion >5 mm. • Lumbosacral spondylolisthesis >2 mm. • Lumbosacral spondylolysis. • Lumbosacral facet arthrosis and/or effusion with correlating facet-mediated pain. • Beck Depression Inventory (BDI) score >24. • More than 3 Waddell’s signs, presence of compensated injury, or involvement in litigation. • Use of extended-release opioid analgesics with associated “addictive behaviors.”* • Body mass index (BMI) >40.* • Functional disability or neurological condition preventing early mobility after BVNA.

⁎ Not an exclusion criterion for the single-arm study.

**Table 2:** Modic change classification based on MRI signal characteristics and clinical implications

Modic Change	Type 1	Type 2	Type 3
T1 weighted MRI description	Hypointense signal	Hyperintense signal	Hypointense signal

T1 MRI pictorial			

T2 weighted MRI description	Hyperintense signal	Intermediate intensity signal	Hypointense signal

T2 MRI pictorial			

Implication	Marrow inflammation and edema	Marrow with fatty replacement	Micro-fractures and sclerosis

Summary of the 3 types of Modic changes, highlighting their characteristics on T1 and T2 weighted MRI scans in addition to their clinical implications. Type 1 Modic changes reflect inflammation and edema in the bone marrow, Type 2 Modic changes indicate fatty replacement of marrow, and Type 3 Modic changes are associated with sclerosis and micro-fractures. Pictorial of hypointense and hyperintense patterns for each Modic change classification displayed below description. (Table adapted from Basivertebral Nerve Ablation: Procedure Overview by Jeffery M Epstein, MD (https://aanos.org/wp-content/uploads/2023/06/Jeff-Epstein-Basivertebral-Nerve-Ablation.pdf); Pictorial adapted from (https://www.researchsquare.com/article/rs-56740/v1).

A 2022 pooled cohort design study by Boody et al. evaluated 296 patients across 3 prospective trials, 2 randomized controlled trials (RCTs) and 1 single-arm prospective study, studying vertebrogenic cLBP in the presence of Type 1 and/or Type 2 Modic changes as well as anterior spinal pain patterns [7]. Analysis found cLBP lasting at least 5 years and higher Oswestry Disability Index (ODI) scores prior to BVNA may lead to better treatment outcomes [7]. While a longer duration of pain traditionally correlates with greater challenge in management, the investigators postulated that these patients were more likely to experience true/dominant vertebrogenic cLBP that had gone unrecognized and untreated. Conversely, opioid use and elevated Beck Depression Inventory (BDI) scores predicted lower BVNA response rates. Despite these findings, no factors beyond the baseline criteria consistently predicted outcomes, likely due to already high response rates within this selected group.

Subsequent studies investigated whether pain locations and exacerbating factors could refine selection [8,9]. Approximately 71% of BVNA responders reported midline-dominant pain, though many also noted paraspinal (47%) or lateral (52%) pain [8]. Flexion (81%), sitting (79%), and standing (69%) were common exacerbating factors among responders [8]. Despite these trends, a clear positive predictor of treatment success was not identified and only a modest negative association was reported with extension-type pain (OR 0.586). As such, midline-dominant low back pain remains the most reliable clinical predictor when baseline criteria (Table 1) are met.

Additional MRI features such as Modic change height and area, vertebral endplate defect size, number, and shape, and Pfirmann scores have also been assessed for effect on BVNA responder rates [9]. Among these, only facet joint fluid at the treated level significantly predicted lower success (OR 0.585), highlighting the importance of excluding facet-mediated pain, and no factors predicted higher response rates. Thus, Type 1 or Type 2 Modic changes are the most important imaging criteria for BVNA patient selection.

Collectively, these studies reinforce that established inclusion/exclusion criteria (Table 1) lead to high BVNA responder rates. While there is evidence of weak or inconsistent associations, characteristics suggestive of other potential pain generators likely decreased treatment success odds and therefore it may be reasonable to perform additional diagnostic evaluations to assess for alternative pain generators such as facet pain or dynamic instability prior to proceeding with BVNA [43].

In summary, current evidence supports strict selection criteria for optimizing BVNA responder rates. As such, practitioners may benefit from utilizing similar selection criteria to the studies reviewed herein to optimize treatment success with considerations for ruling out other potential etiologies of cLBP. Clinical judgment remains essential, particularly when considering BVNA for patients with mixed pain etiologies or partial alignment with trial criteria. Physicians therefore must use their best judgement in weighing risks and benefits, specific to each unique patient, before offering BVNA. We recommend that physicians maintain transparency during patient education and counseling with regard to the state of evidence on clinical outcomes.

### Clinical outcomes

Multiple studies have evaluated the effectiveness and safety of BVNA, consistently demonstrating clinically meaningful improvements in pain and function with a favorable safety profile.

Pivotal to the introduction of BVNA, the SMART (Surgical Multicenter Assessment of Radiofrequency Ablation for the Treatment of Vertebrogenic Back Pain) trial enrolled 225 patients in the U.S. and Europe with cLBP and Type 1 and/or Type 2 Modic changes on MRI and randomized these participants to receive either BVNA (*n*=147) or a sham treatment (*n*=78) while investigating pain and functional improvements via the Visual Analog Scale (VAS) and ODI, respectively [15,44,45]. This trial served as the United States Food and Drug Administration’s Investigational Device Exemption study. At 3 months, BVNA yielded significantly greater ODI reduction (p=.019), with 75.6% achieving clinically meaningful improvement versus 55.3% in the sham group. VAS scores also showed significant improvement at 6-month (p=.008) and 12-month post-BVNA (p=.038) [44]. Function and pain improvements remained durable long-term with statistically significant reductions to mean ODI (2 year: p<.001; 5 year: p<.016) and VAS scores (2 year: p<.001; 5 year: p<.002) [15,45]. Only 1 device-related adverse event, a vertebral compression fracture in the setting of osteopenia and high dose hormone therapy, was reported in all BVNA participants after 5 years (original randomization and crossover; *n*=225). Similar sustained improvement in pain and physical function have been observed in additional studies including the INTRACEPT RCT [41,[46], [47], [48], [49]], and prospective cohort studies reported on by Truumees et al. [50] and Macadaeg et al. [51]. Some studies have suggested that approximately 10% of BVNA patients may develop radicular pain or sensory deficits following the procedure; however, nearly all these cases are temporary and pedicle breach may have contributed to symptoms [41,44,47,50,52].

The INTRACEPT RCT [41,[46], [47], [48], [49]], a prospective, open-label RCT conducted at 20 sites evaluating BVNA versus standard care in 140 patients (treatment arm: *n*=66; control arm: *n*=74) with cLBP and Modic Type 1 or 2 vertebral endplate changes, further confirmed BVNA efficacy, with durable, superior outcomes. Three months post-BVNA, a mean ODI reduction of 25.3 points compared to 4.4 points in the standard care group (adjusted difference: 20.9, p<.001) and a VAS pain reduction of 3.5 cm versus 1.0 cm (adjusted difference: 2.44 cm, p<.001) were reported. Additionally, 74.5% of the BVNA patients achieved at least 10-point ODI improvement compared to 32.7% in the standard care group (p<.001) at this time. As a result, an independent data monitoring committee recommended that enrollment stop and control arm participants be offered early crossover to BVNA [46]. Sustained benefits continued to be observed at 12 months with a mean ODI and VAS reductions of 25.7 points (p<.001) and 3.8 cm (p<.001), respectively [46]. Further improvements in these metrics were also observed at 24 months (ODI: 28.5 points [p<.001]; VAS 4.1 cm [p<.001]) [47]. Five-year pooled data from INTRACEPT, SMART, and a third cohort showed continued durability and no new serious device-related events [46,49].

Several single-arm studies such as Becker et al. [53], Fishchenko et al. [54], Kim et al. [55], De Vivo et al. [14], Schnapp et al. [56], and Fogel et al. [57] have further supported these findings. Notably, Schnapp et al. [56] reported similar statistically significant improvements in ODI and VAS scores at 3 and 6 months post-BVNA in an older patient population while Fogel et al. [57] specifically included patients with adult degenerative spinal deformity with comparable findings. This latter study did report 9 vertebral compression fractures (10% of their “high-comorbidity” subgroup) in individuals with severe osteoporosis, highlighting the need for osteoporosis screening and potential treatment prior to BVNA [57]. In fact, many recommend osteoporosis be treated for at least 3 months prior to BVNA, loosely extrapolating from spinal fusion literature [58]. Overall, these single-arm studies are largely consistent with higher level evidence derived from aforementioned RCTs, supporting the effectiveness of BVNA in patient populations with modest deviation from original trial criteria.

Numerous systematic reviews and meta-analyses also support the long-term effectiveness and relative safety of BVNA for treating cLBP [40,[59], [60], [61], [62]]. Meta-analyses indicate that approximately 65% and 75% of patients experience clinically meaningful pain and functional improvements at 6 and 12 months, respectively, with stable results as far as 60 months [40,59,60]. Of note, clinical outcomes of BVNA were superior to those associated with intradiscal steroid injections, pulsed radiofrequency ablation, and annuloplasty in pain and function improvement at 6 and 12 months in similar patient populations [60]. While biologic therapies and multifidus muscle stimulation showed benefits at 6, 12, and 24 months, BVNA had significantly fewer serious adverse events compared with these interventions. Specifically, multifidus stimulation studies reported a 23.5% rate of surgical revisions within 2 years, whereas BVNA studies reported no serious device or procedure-related adverse events [60].

Such results have informed broader guidelines for care of cLBP. Namely, the North American Spine Society (NASS) and numerous other organizations have declared BVNA medically necessary for patients with cLBP that has not improved with conservative treatment and shows vertebrogenic pain with evidence of Type 1 or Type 2 Modic changes on MRI [63]. The NASS coverage policy for BVNA supports vertebrogenic back pain as a distinct condition and recognizes that BVNA has been shown to significantly improve pain and function over standard care [64]. NASS recommends BVNA for skeletally mature patients with cLBP lasting over 6 months, who have failed nonoperative treatment and have appropriate imaging biomarkers on MRI. Of note, BVNA is not recommended for those with other spinal pathologies, metabolic bone disease, trauma, cancer, or infection. These NASS guidelines ensure appropriate use, aligning treatment with the latest evidence in vertebrogenic pain management.

### Low back pain-related healthcare utilization and cost-effectiveness

BVNA has been associated with reduced low back pain-related healthcare utilization (LBPr-HU) and favorable cost-effectiveness. McCormick et al. analyzed LBPr-HU before and after treatment with BVNA in a pooled cohort study, incorporating data from 3 prospective clinical trials (SMART, INTRACEPT, and the Truumees et al. single-arm prospective cohort study) [65]. The study assessed LBPr-HU data from 247 patients who underwent BVNA and had follow-up at a minimum of 1 year. Of these 247 patients, 205 had long-term follow-up (mean 5.3 years post-BVNA), at which time LBPr-HU was also assessed. The categories of LBPr-HU included noninvasive conservative care (physical therapy, chiropractic care, acupuncture), opioid utilization, lumbosacral spinal injections (LSI), lumbosacral radiofrequency ablation (LRFA), and lumbosacral spinal surgery. Investigators found a 27% reduction in patients using conservative care in the year following BVNA. There were significant reductions in opioid use, with 40.3% fewer patients using opioids at 1 year and 61.7% fewer at long-term follow-up. LSI use decreased by 81.2% after 1 year and this reduction remained similar at long-term follow-up. The rates of LRFA utilization were 1.6% at 1 year and 8.3% at long-term follow-up. Lumbar fusion surgery rates were 0.8% at 1 year and 6.5% at long-term follow-up. The authors concluded that BVNA significantly reduced LBPr-HU over 5 years, particularly in terms of conservative care, opioid use, and LSIs, with lumbar fusion rates also lower than expected compared to historical comparisons [[66], [67], [68]].

Smuck et al. also evaluated the cost-effectiveness of BVNA compared to standard care for patients with vertebrogenic cLBP [69]. The analysis was based on data from the same 3 foundational studies (SMART, INTRACEPT, and Truumees et al. single-arm prospective cohort study). The primary outcome measures were costs and quality-adjusted life years (QALYs), with the incremental cost-effective ratio (ICER) calculated to determine cost-effectiveness. Quality of life measures were assessed using the EuroQol Group 5 Dimension 5-Level (EQ-5D-5L) questionnaire and SF36v2®. The base case analysis demonstrated that BVNA was a cost-effective treatment strategy compared to standard care, with an ICER of $11,376 per QALY over a 5-year horizon. BVNA was highly cost-effective, with a greater than 99% probability of meeting the cost-effective threshold of $100,000 to $150,000 per QALY. Sensitivity and scenario analyses supported these findings, showing consistent ICERs below this threshold. The authors concluded that BVNA alone is a cost-effective treatment for vertebrogenic cLBP compared to standard care alone.

## Conclusions

As spine care moves towards precision-medicine with improved identification of cLBP phenotypes and development of condition-specific interventions, physicians must understand the phenomenon of vertebrogenic cLBP and when a patient with this diagnosis may be a candidate for treatment with BVNA. We recommend using similar selection criteria to the randomized clinical trials discussed here to optimize treatment success rates with appropriate clinical consideration made to rule out other etiologies with distinct treatment requirements such as facet pain or dynamic instability. Patient-specific scenarios arise in which it may be reasonable to proceed with BVNA, even if some of the ideal selection criteria are not met.

The current body of evidence including randomized controlled trials, prospective cohort studies, and observational studies indicates efficacy, effectiveness, relative safety, reduced LBPr-HU, and cost-effectiveness associated with BVNA used for the treatment of vertebrogenic cLBP. These outcomes in a majority of treatment responders appear to be durable for over 5 years post-BVNA when the original randomized clinical trial inclusion and exclusion criteria are followed. Healthcare utilization and economic analyses demonstrate that BVNA significantly reduces healthcare utilization, particularly opioid utilization, spinal injections, and surgery rates with potential long-term cost savings in a similar population. Model-based evaluations establish BVNA as a highly cost-effective strategy compared to standard care, with an incremental cost-effectiveness ratio well below standard thresholds.

Finally, given the narrative nature of this review, there are poignant limitations to these conclusions. Firstly, the generalizability of randomized trial findings may be restricted by the strict inclusion/exclusion criteria that may not best reflect real-world populations. Additionally, long-term outcomes in patients with more complex or comorbid spinal conditions remain underexplored. Future research should focus on refining imaging biomarkers for vertebrogenic pain, identifying predictors of treatment response across broader populations, and evaluating the effectiveness and safety of BVNA in patients with overlapping spinal pathologies. Comparative studies of BVNA against newer interventions and stratified cost-effectiveness analyses will also be critical as spine care evolves.

## Author contribution

RE and JB contributed equally as co-first authors to all aspects of the project, including conceptualization, methodology, data curation, formal analysis, visualization, drafting of the manuscript, and critical review and editing. BS and ZM contributed to conceptualization, data curation, and provided revisions and critical feedback during manuscript review and editing.

## Declarations of competing interests

The authors declare that they have no known competing financial interests or personal relationships that could have appeared to influence the work reported in this paper.
